# Pitfalls in the management of isolated pulmonary Takayasu’s arteritis after surgery: a case report of an experience during 34 months after a pulmonary artery graft replacement

**DOI:** 10.1186/s13019-016-0413-3

**Published:** 2016-01-16

**Authors:** Kishu Fujita, Shu Kasama, Masahiko Kurabayashi

**Affiliations:** Department of Cardiovascular Surgery, Hokkaido Ohno Hospital, 1-1-30 Nishino 4 Jyou, Chuo-ku, Sapporo, Hokkaido, 063-0034 Japan; Department of Medicine and Biological Science (Cardiovascular Medicine), Gunma University Graduate School of Medicine, 3-39-22 Showa-machi, Maebashi, Gunma 371-8511 Japan

**Keywords:** Takayasu’s arteritis, Pulmonary artery surgery, Postoperative course

## Abstract

**Background:**

Several controversial matters still remain unresolved in the management of Takayasu’s arteritis, especially after vascular intervention. First, a definitive diagnostic tool has not been established to assess disease activity correctly. Second, the optimal medical regimen has not been established to prevent restenosis of the vascular lesion.

Surgical treatments have been rarely performed to relieve critical vascular stenosis in isolated pulmonary Takayasu’s arteritis, but their postoperative courses on long-term follow-up periods have not been sufficiently reported.

**Case Presentation:**

A 48-year-old man underwent a successful graft replacement for severe right main pulmonary artery stenosis due to isolated pulmonary Takayasu’s arteritis. The patient had remained asymptomatic with no clinical inflammatory signs under adequate anticoagulation therapy since then. However, stenosis of the prosthetic graft accompanied by marked pulmonary hypertension was detected 18 months after surgery. Anti-inflammatory treatment with only 5 mg/day of oral prednisolone was then implemented, and the stenosis remained unchanged with the patient being stable for the next 16 months.

**Conclusions:**

This is the first published case report that describes the actual clinical course with a long-term follow-up period after surgery for isolated pulmonary Takayasu’s arteritis, including images of the stenosed prosthetic graft.

This case suggests that patients should be followed with multiple complementary diagnostic techniques on the assumption that restenosis is highly possible and unpredictable even after surgery. Besides, sufficient anti-inflammatory treatment should be applied as soon as possible after surgery no matter how inactive the disease appears to be, although its optimal regimen especially during the inactive inflammatory phase needs to be further established.

## Background

Surgical treatments have been performed, albeit rarely, to relieve critical vascular stenosis in isolated pulmonary Takayasu’s arteritis (TA), a very rare TA subtype which primarily involves pulmonary arteries (PAs) without any systemic arterial lesions [1–4]. To our knowledge, 13 case reports, including our own report, have described 3 different types of surgical procedures: patch angioplasty in 5 cases, bypass surgery accompanied with no resection of the involved lesions in 2 cases, and graft replacement accompanied with removal of the pathologic vessels in 6 cases. The surgical outcomes were satisfactory with no mortality and no serious complications [1] (Table 1).

**Table 1:** Summary of the surgery for isolated pulmonary TA

Pt	Age (Y), gender	Location	Procedure	Material applied	Follow-up months	Postoperative imaging	Restenosis	Immuno- suppression	Author (’Y)
1	60 F	Bifurcation	Patch	Pericardium	6	AG	Yes	Yes*	Chauvaud (’87)
2	53 M	Bifurcation	Patch	Pericardium	6	AG	Yes	-	Dietl (’88)
3	57 M	Rt	Patch	-	-	-	-	-	Okubo (’88)
4	31 M	Bifurcation	Patch	Dacron	18	AG	Yes	-	Jakob (’90)
5	34 F	Rt	Grafting	NA	13	-	-	-	Lie (’96)
6	25 F	Rt	Grafting	NA	25	-	-	-	Lie (’96)
7	76 F	Rt & Lt	Bypass	PTFE	-	-	-	-	Sundt (’01)
8	63 F	Rt & Lt	Grafting	Dacron	66	-	-	-	Shikata (’04)
9	67 F	Rt	Patch	Pericardium	3	AG	Yes	-	Yamazaki (’05)
10	45 F	Lt	Bypass	PTFE	60	AG	No	-	Nakajima (’07)
11	48 M	Rt	Grafting	Dacron	6	CT	No	No	Fujita (’13)
12	59 M	Rt & Lt	Grafting	PTFE	1.5	AG & CT	No	Yes^†^	Hamamoto (’11)
13	51 F	Rt & Lt	Grafting	PTFE	30	AG & CT	No	-	Furtado (’12)

Pt: patient number, Y: year, M: male, F: female, Bifurcation: bifurcation of the main pulmonary artery trunk, Rt: right main pulmonary artery, Lt: left main pulmonary artery, Patch: patch angioplasty, Grafting: graft replacement, Bypass: bypass surgery, PTFE: polytetrafluoroethylene, AG: angiography, CT: computed tomography, *: glucocorticoid at unknown dosage, ^†^: oral prednisolone at 20 mg/day, −: not available

However, the postoperative courses on long-term follow-up periods have not been sufficiently reported, although TA is basically a chronic, progressive, and relapsing disease and restenosis of the vascular lesion occurs quite commonly [3, 5]. In 11 of those 13 cases, the follow-up results were mentioned with relatively short follow-up periods from 1 to 66 months (21.3 ± 21.5 months), and only 8 patients underwent imaging studies, e.g. either computed tomography (CT), magnetic resonance imaging, or angiography to evaluate the patency of the surgical materials [1]. These imaging studies revealed mild restenoses at the surgical sites without clinical signs or symptoms in 4 of the patients at as early as 3 months postoperatively. Those 4 cases were all managed by patch angioplasty at the PA bifurcation, leaving the native vessel wall behind. No details on their clinical courses after restenoses were described, and also no actual images of the restenoses were presented (Table 1).

## Case Presentation

**Fig. 1:**
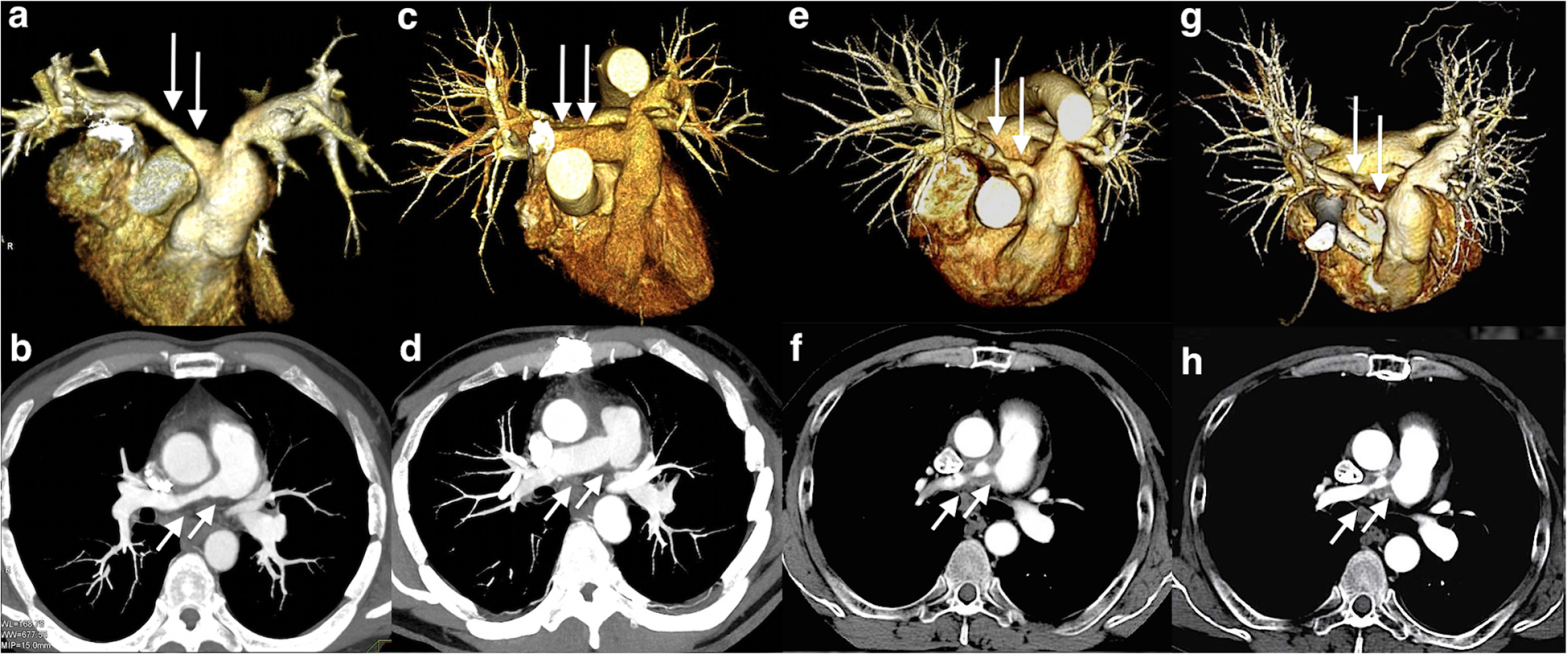
Chest CT images: (**a**, **b**) Before surgery, showing stenosis of the right PA (arrows); (**c**, **d**) At 6 months after surgery, showing the patent grafts of the right PA (arrows); (**e**, **f**) At 18 months after surgery, showing the stenosis of the right PA graft (arrows); (**g**, **h**) At 34 months after surgery, showing unchanged stenosis of the right PA graft (arrows)

**Fig. 2:**
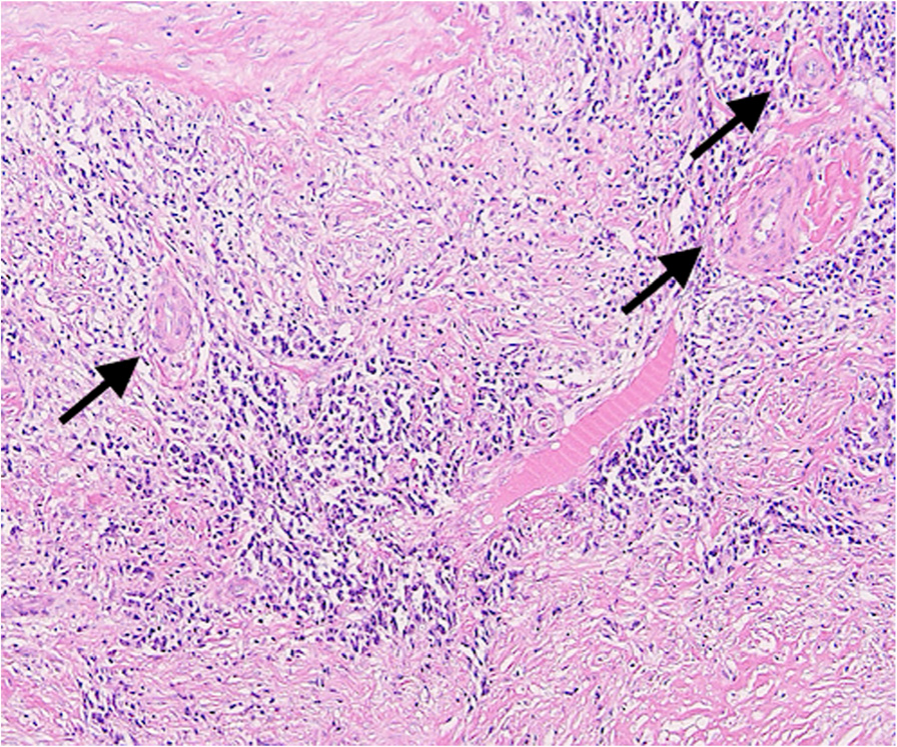
A section of the right PA, showing granulomatous lymphoplasmacytic arteritis of the media with irregularly distributed Langhans’ giant cells (arrows, hematoxylin and eosin, 100x)

The patient had severe stenosis of unknown etiology in the middle of the right main PA, accompanied by marked pulmonary hypertension and dysfunction of the right ventricle (RV) at age 48 (Fig. 1a, b). The existing hemodynamic strains required endovascular or surgical intervention to relieve the stenosis, and we chose surgery, hoping to establish the diagnosis. We were concerned about either local progression or spread to the other area of this undiagnosed stenosis if the lesion was left behind with patch angioplasty or bypass surgery. Thus, we decided to perform graft replacement with total excision of the lesion, and an 18 mm Dacron graft (Triplex^R^, Terumo Corporation, Japan) was anastomosed in an end-to-end fashion behind the ascending aorta. He was finally diagnosed as having isolated pulmonary TA only by the pathological examination of the resected right PA (Fig. 2), and began anticoagulation therapy with warfarin to attain a target prothrombin time - international normalized ratio (PT-INR) of 2.0 – 2.5 in addition to aspirin at 100 mg/day. At the 6-month follow-up after surgery, enhanced chest CT showed a patent graft (Fig. 1c, d), and transthoracic echocardiography (TTE) demonstrated normalized RV function [1]. The patient remained asymptomatic since then, and was followed with laboratory tests every 2 months, which showed no remarkable changes in C-reactive protein (CRP) values of less than 0.5 mg/dl. He had TTEs every 6 months, which demonstrated no abnormality at his 12-month follow-up (Table 2).

**Table 2:** Pre- and postoperative laboratory and TTE findings

	Pre	6M	12M	18M*	24M	31M^†^	34M
Laboratory findings							
WBC (/μL)	7200	3900	6700	7100	4800	6200	2900
CRP (mg/dL)	0.26	0.18	0.16	0.35	0.09	0.21	0.26
ESR (mm/1hr)	10	6	-	1	-	-	-
PT-INR	0.99	2.09	2.42	2.42	1.85	2.27	2.13
TTE findings							
TRPG (mmHg)	38	50	ND	77	64	30	71
RVSP (mmHg)	41	53	ND	80	72	33	74
PAPG (mmHg)	-	-	-	-	63	45	52
Tr	Trivial	Trivial	None	Trivial	Trivial	Trivial	Trivial
EF (%)	67	63	59	69	64	71	61

TTE: transthoracic echocardiography, Pre: preoperatively, M: months after surgery, *: detection of the restenosis, ^†^: at readmission, WBC: white blood cell, CRP: C-reactive protein, ESR: erythrocyte sedimentation ratio, PT-INR: prothrombin time-international normalized ratio, TRPG: tricuspid regurgitation pressure gradient, RVSP: right ventricular systolic pressure, PAPG: pressure gradient between main and right pulmonary artery, Tr: tricuspid regurgitation, EF: ejection fraction, ND: not detected, −: not available

**Fig. 3:**
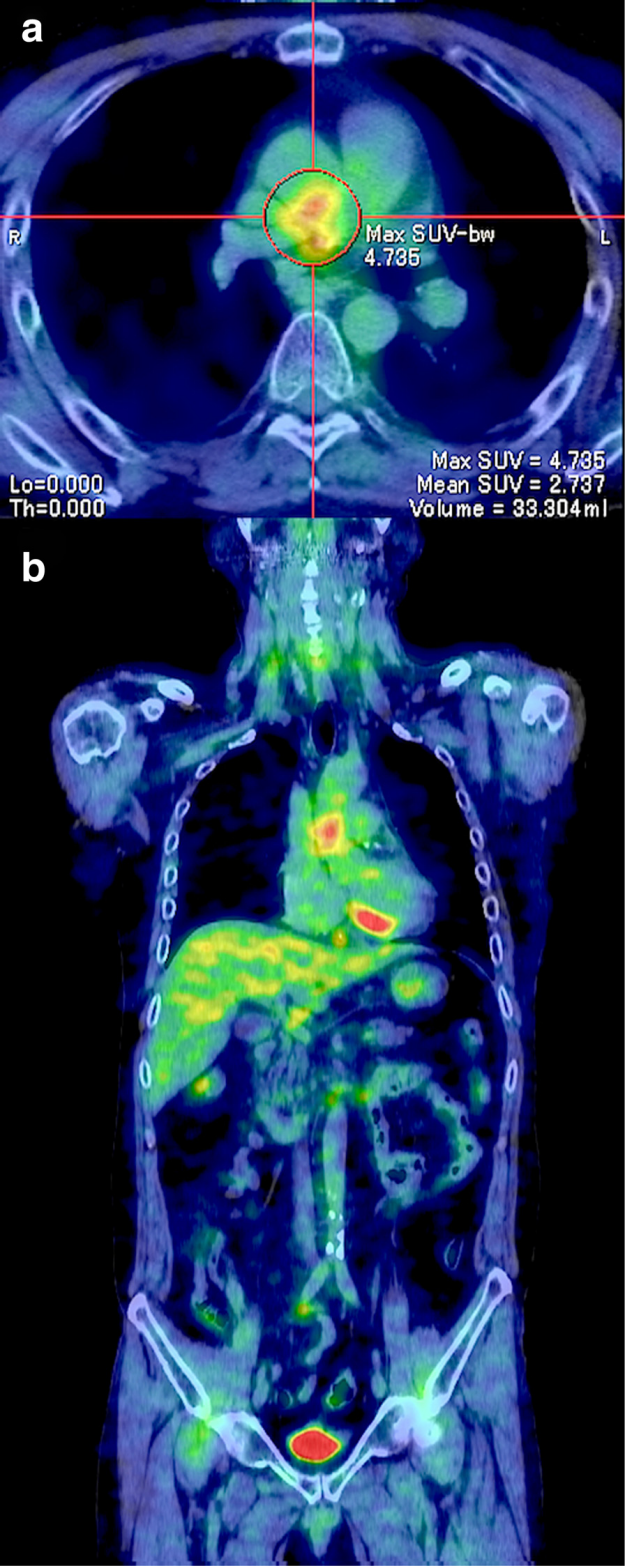
FDG-PET CT images at 18 months after surgery: (**a**) Abnormal uptakes were detected only around the right PA graft; (**b**) No other apparent abnormal uptake was detected on the other large vessels

However, TTE at the 18-month follow-up showed remarkable pulmonary hypertension and RV dysfunction with preserved left ventricle systolic function (Table 2). Thus, enhanced chest CT was performed and revealed severe stenosis of the right PA graft especially at the anastomotic sites, surrounded by a circumferential abnormal shadow covering the full length of the graft (Fig. 1e, f). Peripheral PAs distal to the PA graft were intact, and no other apparent abnormalities or changes on the other chest and neck vessels were observed when compared to that of the previous studies. Whole body positron emission tomography with fluorine-18 fluorodeoxyglucose CT (FDG-PET) was then performed for the first time and demonstrated high FDG uptake only around the right PA graft, but no other abnormal uptake by the other vessels was observed (Fig. 3a, b). The FDG-PET result suggested that focal inflammation around the graft was the most probable cause of the stenosis, although CRP levels remained almost unchanged (Table 2).

Oral prednisolone at 10 mg/day was thus initiated, but was reduced to 5 mg/day 2 months afterwards because of its potential side effects, including facial edema, depressive mood disorder, and mild liver dysfunction. The facial edema persisted since then, but otherwise the patient remained asymptomatic and did not consent to take additional immunosuppressive agents. Repeated CTs, TTEs, and laboratory tests at 24- and 30-month follow-ups demonstrated no remarkable changes (Table 2).

However, the patient gradually complained of dyspnea on exertion since then. An oral diuretic was added on suspicion of exacerbating right heart strain, but the patient was readmitted to the hospital due to worsening symptoms 31 months after surgery. TTE on re-admission revealed no remarkable change compared to that of the previous studies, except for decreased right ventricular systolic pressure, which seemed to be inconsistent with right heart failure but rather indicated hypovolemia (Table 2). Indeed, cessation of the diuretic and fluid replacement improved the patient’s condition and symptoms rapidly. Re-catheterization and subsequent angioplasty or re-surgery were thus offered, but the patient refused any interventional procedures. The patient also refused increasing the dose of prednisolone, complaining of the still persisting facial edema.

After leaving the hospital, the patient’s physical condition remained stable on the same dosage of prednisolone. CT and TTE performed at the 34-month follow-up, 3 months after re-admission, showed no remarkable change in the right PA graft stenosis and similar pulmonary hypertension. Thus, the stenosis accompanied with pulmonary hypertension and RV dysfunction was almost unchanged for as long as 16 months since its first detection (Fig. 1g, h, and Table 2). Thereafter, contact with the patient was lost.

## Discussion

We can define 3 clinical pitfalls in this case, although the definite etiology of the graft stenosis remained unestablished and a longer follow-up period with repeated assessment of the local inflammatory activity was desirable.

First, we should have taken into account the fact that the inflammatory process in TA can remain active at a subclinical level, no matter how inactive it appears to be [4, 5, 7]. In addition, identifying such subclinical inflammation prior to subsequent recurrence is very difficult, because clinical, laboratory, and radiologic data do not always correlate with each other [2–7]. CRP and erythrocyte sedimentation rate, which are generally used as safe and non-invasive inflammatory surrogate markers, are not always reliable [7–9]. Indeed, the stenosis of the right PA graft had presumably progressed between 12 and 18 months after surgery in this case, but neither clinical features nor laboratory tests alone could demonstrate this on-going stenosis. Thus, CT was not performed after the 6-month follow-up, but if we had performed imaging studies with either CT or magnetic resonance imaging periodically, regardless of whether any signs for stenosis were perceived or not, the stenosis might have been detected at an earlier stage [9]. Moreover, FDG-PET would have been of value for monitoring [9]. It could be useful especially when the assessment of disease activity is difficult due to the absence of clinical features with absent or low CRP elevation, because it can detect even subtle inflammatory activity in the vessel walls with higher sensitivity than CRP [4, 7]. If we had performed FDG-PET soon after the surgery to examine the actual inflammatory activity around the PAs, it might have encouraged us to commence anti-inflammatory treatment earlier, although the other clinical findings were incompatible with active inflammation.

Second, anti-inflammatory treatment, starting no later than the establishment of the pathological diagnosis of TA, might have altered the clinical course [2, 6]. The graft stenosis could occur due to any reason, especially with pannus formation or thrombosis, but the FDG-PET result suggested that the local inflammation was involved in the graft stenosis. We did not initiate immunosuppression until discovery of the restenosis, assuming that the graft replacement with entire resection of the pathological PA wall could deprive the vasculitis of its normal course of action. The fact that systemic inflammatory features were absent perioperatively and that the above-mentioned 4 published cases with late restenoses were all managed by patch angioplasty leaving the pathological PA wall behind seemed to concur with our speculation. But the total removal of the pathological right PA wall was not guaranteed even with the pathological examination of the surgical specimen. Moreover, the absence of systemic inflammatory features should not have omitted immunosuppression, because systemic clinical manifestations are usually rare or absent in isolated pulmonary, in which the inflammatory process involves only PAs [3, 4]. Besides, reliable and timely evaluation of disease activity is difficult, and established vascular stenosis cannot usually be reversed by medical treatment alone [2, 3, 6]. Therefore, anti-inflammatory treatment should have been considered as soon as possible after surgery, regardless of the apparent clinical inflammatory status [2].

Finally, another anti-inflammatory regimen with either a higher-dose of glucocorticoids or combined with other immunosuppressants might have resulted in a different clinical course [2, 3, 6]. The current European League Against Rheumatism (EULAR) recommendation proposes an aggressive induction therapy for the management of large vessel vasculitis, consisting of an initial 1 mg/kg oral prednisolone dose (maximum 60 mg/day) for one month with subsequent tapering to 10–15 mg/day for several months [9]. However, this recommendation does not mention in which condition this regimen should be applied. This patient’s clinical condition at the commencement of glucocorticoids was not suggestive of active inflammatory status, which caused us to hesitate in applying such a high dose of glucocorticoids. Attendant adverse effects of them, particularly either increased thrombogenecity or risk of infection, cannot be ignored especially immediately after surgery [2, 4]. Actually, even such a low initial dose, oral prednisolone of 10 mg/day in this 72 kg-patient, was reduced to 5 mg/day due to its known adverse effects. But we could have at least performed FDG-PET repeatedly and considered additional immunosuppressive agents, which can help to improve disease control and facilitate reduction of the cumulative glucocorticoid dose [2, 6, 9]. Besides, there are a few case reports of isolated pulmonary TA, which demonstrated decreased PA pressure after anti-inflammatory treatment with high dose of glucocorticoids and additional immunosuppressive agents, despite of unchanged stenotic lesions in imaging studies [6, 10]. Therefore, even after the development of significant restenosis, sufficient anti-inflammatory treatment might have been worthwhile, although its optimal regimen has yet to be defined.

## Conclusion

This case suggests that patients after surgery for isolated pulmonary TA should be followed with multiple complementary diagnostic techniques, including CT, magnetic resonance imaging, and in particular, FDG-PET at regular intervals, on the assumption that restenosis is highly possible and unpredictable. Anticoagulation therapy alone is insufficient to prevent restenosis, and sufficient anti-inflammatory treatment should be applied as soon as possible after surgery regardless of the apparent inflammatory status, although the optimal regimen needs to be further established.

## Consent

Written informed consent was obtained from the patient during the second hospitalization for publication of this Case report and any accompanying images. A copy of the written consent is available for review by the Editor-in-Chief of this journal.

## Abbreviations

TA: Takayasu’s arteritis
PA: Pulmonary arteries
CT: Computed tomography
RV: Right ventricle
TTE: Transthoracic echocardiography
CRP: C-reactive protein
FDG-PET: Positron emission tomography with fluorine-18 fluorodeoxyglucose CT
EULAR: European League Against Rheumatism

## References

[1] Fujita K, Nakashima K, Kanai H, Kumakura H, Minami K (2013). A successful surgical repair of pulmonary stenosis caused by isolated pulmonary Takayasu’s arteritis. Heart Vessels.

[2] Park MC, Lee SW, Park YB, Lee SK, Choi D, Shim WH (2006). Post-interventional immunosuppressive treatment and vascular restenosis in Takayasu's arteritis. Rheumatology.

[3] Qin L, Hong-Liang Z, Zhi-Hong L, Chang-Ming X, Xin-Hai N (2009). Percutaneous transluminal angioplasty and stenting for pulmonary stenosis due to Takayasu's arteritis: clinical outcome and four-year follow-up. Clin Cardiol.

[4] Vista EG, Santos Estrella PV, Lichauco JJ (2010). Flourine-18 flourodeoxyglucose positron emission tomography as a non-invasive test of disease activity in Takayasu's arteritis-a report of four cases. Autoimmun Rev.

[5] Maksimowicz-McKinnon K, Clark TM, Hoffmann GS (2007). Limitations of therapy and a guarded prognosis in an American cohort of Takayasu arteritis patients. Arthritis Rheum.

[6] Toledano K, Guralnik L, Lorber A, Ofer A, Yigla M, Rozin A (2011). Pulmonary arteries involvement in Takayasu's arteritis: two cases and literature review. Semin Arthritis Rheum.

[7] Ishihara T, Haraguchi G, Tezuka D, Kamiishi T, Inagaki H, Isobe M (2013). Diagnosis and assessment of Takayasu arteritis by multiple biomarkers. Circ J.

[8] Hoffman GS, Ahmed AE (1998). Surrogate markers of disease activity in patients with Takayasu arteritis. A preliminary report from The International Network for the Study of the Systemic Vasculitides (INSSYS). Int J Cardiol.

[9] Mukhtyar C, Guillevin L, Cid MC, Dasgupta B, de Groot K, Gross W (2009). EULAR recommendations for the management of large vessel vasculitis. Ann Rheum Dis.

[10] Fukuda Y, Shirai K, Takamiya Y, Nathan M, Mito T, Yamagi D (2008). Isolated pulmonary arterial stenosis caused by Takayasu's arteritis in an elderly male. J Cardiol.

